# Global and Indian research trends on Yoga: Insights from WHO trial registry

**DOI:** 10.6026/973206300210030

**Published:** 2025-01-31

**Authors:** Himel Mondal, Satya Lakshmi Komarraju, Sathyanath D., Shrikanth Muralidharan

**Affiliations:** 1 Department of Physiology, All India Institute of Medical Sciences, Deoghar, Jharkhand - 814152, India; 2 Director, National Institute of Naturopathy, Ministry of Ayush, Government of India, Pune - 411001, India; 3 National Institute of Naturopathy, Ministry of Ayush, Government of India, Pune - 411001, India; 4 Department of Research, National Institute of Naturopathy, Ministry of Ayush, Government of India, Pune - 411001, India

**Keywords:** Yoga, clinical trials, research trends, world health organization (WHO), international clinical trials registry Platform (ICTRP), intervention, India, chronic diseases, mental health

## Abstract

A retrospective analysis of 2919 yoga-related clinical trials registered in the World Health Organization (WHO) International Clinical Trials Registry Platform database using the keyword "yoga" on 10 September 2024 is of interest. Most trials were interventional (97.81%) and registered in the Clinical Trials Registry of India (56.39%) and ClinicalTrials.gov (26.07%). The majority included both genders (69.29%), with a mean age range of 23.9±13.45 to 56.21±24.66 years, focusing on chronic diseases like obesity, diabetes, hypertension and mental health conditions such as anxiety and depression. There was a steady increase in registrations over the years, reflecting the growing global interest in yoga research. Thus, the use of yoga as a therapeutic tool to support its integration into healthcare systems is highlighted.

## Background:

Yoga, an ancient practice with origins in India, has evolved into a holistic approach to health that extends far beyond physical exercise [1]. Its integration of body, mind and spirit has made yoga a popular method for managing lifestyle-related diseases such as hypertension, diabetes and heart disease [2]. Increasingly, yoga is being adopted worldwide as a complementary therapy, offering both preventive and therapeutic benefits for physical and mental well-being [3, 4]. As yoga becomes more prominent in healthcare, research on its effects is critical. Scientific studies are necessary to validate its benefits and explore its potential in various medical conditions [5]. Evidence-based research helps integrate yoga into clinical practices, shaping public health strategies and patient care. Without rigorous research, the full potential of yoga in modern medicine may remain untapped [6]. Understanding current trends in yoga research is essential for researchers, policymakers and healthcare providers [7]. By knowing where research is concentrated and identifying gaps, stakeholders can prioritize funding, develop relevant policies and create effective interventions [8]. This data audit of yoga trials, with a focus on both global and Indian research, offers valuable insights into the on-going scientific exploration of yoga, helping guide future research and investment in the field.

## Methods:

This study was a retrospective observational data audit. Data were sourced from the World Health Organization (WHO) International Clinical Trials Registry Platform (ICTRP). This database consolidates clinical trials from national and regional registries. We collected clinical trial data from ICTRP on 10 September 2024. The registry provides a rich source of information on on-going and completed clinical trials. A systematic search was conducted using the keyword "yoga" to identify relevant trials. All the data retrieved with the keyword was saved in comma separated value. Then the data were analyzed in a spreadsheet (Microsoft Excel 2021). Before starting the data analysis, incomplete or erroneous entries were removed from the final analysis. Two individual authors (HM and SM) searched the database and cleaned the data to get their final versions and a consensus was reached to finalize the spread sheet to analyze. Once the relevant trials were identified, key trial information was extracted and compiled into a structured format. This information included the registration date (for finding the trend over time); country, study design, sample sizes and their sex and age range and registering database were recorded. The disease of interest was identified by generating a word cloud. Descriptive statistics were used to summarize the distribution of yoga-related trials globally and in India. The continuous data were expressed in mean and standard deviation with median and quartile range for a better understanding of the data distribution. Categorical data were expressed in number and percentage. For statistical test, we used GraphPad Prism 9.5.0 (GraphPad Software, Boston, USA). A P <0.05 was considered statistically significant. This study was based entirely on publicly available data from the WHO ICTRP and no human subjects or sensitive data were involved. As a result, no formal ethical approval was required for conducting this data audit. All data were handled according to the applicable guidelines for using publicly available information.

## Results:

**Figure 1:**
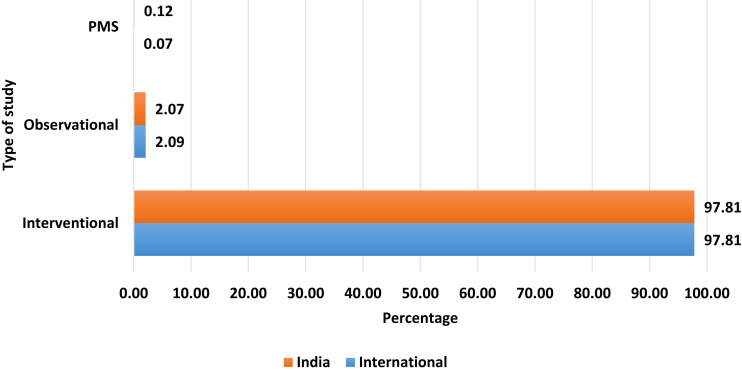
Percentage of studies according to type of study. PMS: post-marketing surveillance

We found a total of 2935 trials in the database. With initial screening, 16 trials were excluded due to incomplete data, yielding 2919 data for analysis. The majority of the global trials were interventional (2855 trials, 97.81%), followed by observational studies (61 trials, 2.09%). India also sowed similar pattern as shown in Figure 1.

**Figure 2:**
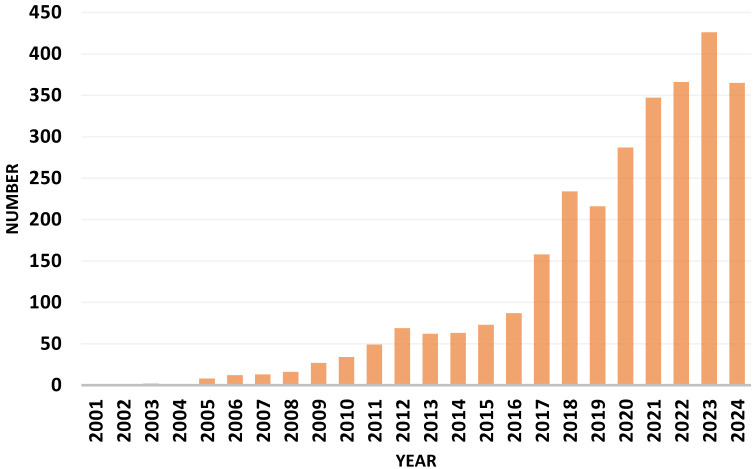
Registered clinical trial according to year of registration from 2000 in WHO ICTRP

A total of 1231 trial provided data on the sex of the participants. The majority of the trials included both male and female participants (853 trials, 69.29%), while 354 trials (28.76%) focused exclusively on female participants and 24 trials (1.95%) included only male participants. In the trials, the mean minimum age of participants was 23.9±13.45 years, while the mean maximum age was 56.21±24.66 years. The majority of yoga-related trials were registered in the Clinical Trials Registry of India (CTRI) with 1,646 trials (56.39%), followed by ClinicalTrials.gov with 761 trials (26.07%). Registries and their number of trials are shown in Table 1. Yoga-related clinical trials have shown in Figure 2 there was a steady upward trend in registration over the years, beginning with just 1 trial in 2001. This growth of number remained low, with a spurt in 2017 with 158 trials. The number of trials peaked in 2023 with 426 registrations. The registration is still continuing and the data we obtained is up to 10th September 2024. Till this date in 2024, 365 trials have already been registered.

**Figure 3:**
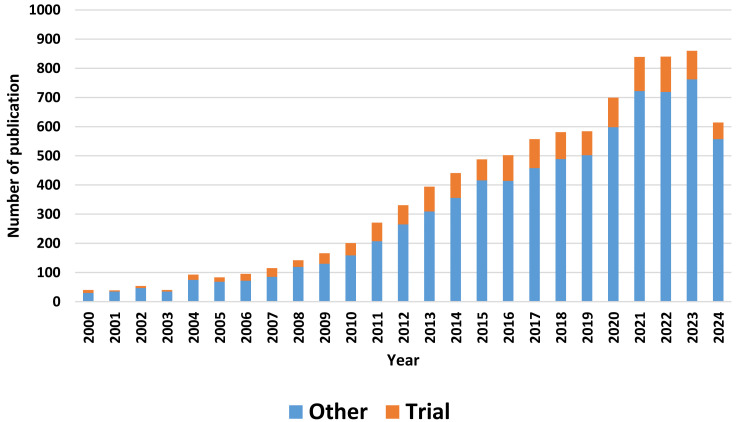
Year-wise publication according to category of the study indexed in PubMed

A word cloud was generated from the expected outcome to get the most common disease where yoga is being used as an intervention. The word cloud is available from the DOI: 10.6084/m9.figshare.28296041. It prominently features chronic diseases like obesity, diabetes, hypertension, alongside mental health conditions such as stress, anxiety and depression. There were studies related breast diseases and cancer. When we searched the publication in PubMed with keyword "yoga", it yielded a total of 8696 publication. Year-wise publication in PubMed is shown in Figure 3. Total percentage of clinical trial was 14.72%. Systematic review and meta-analysis were 10.75% and others were 74.53%.

## Discussion:

The results of this study reveal a strong global focus on interventional yoga trials, with most studies including both male and female participants and a broad age range. India emerged as the primary hub for yoga research, with a substantial number of trials registered in its national registry. Additionally, there has been a notable upward trend in yoga-related trial registrations over the last two decades, reflecting increasing global interest in yoga's therapeutic potential. Chronic diseases such as diabetes, cardiovascular conditions and mental health disorders like anxiety and depression were key areas of focus in these studies. However, yoga-related publication shows very few clinical trials indicating the publication is related to other types of studies and reviews. The dominance of interventional studies establishes concrete evidence of current research on yoga's efficacy in treating or preventing various conditions [9]. Yoga's popularity as a holistic therapy has grown with the rise of lifestyle diseases, prompting researchers to rigorously assess its impact in a controlled manner [10]. India's leading role in yoga research can be attributed to the practice's cultural significance, government initiatives promoting traditional medicine and the country's active participation in clinical trials [11]. Globally, the findings of this study highlight the need to expand research into less explored regions and demographics, ensuring a more comprehensive understanding of yoga's effects across diverse populations and integration with modern medicine [12]. A previous study by McCall found that yoga-related publications on PubMed remained low until 2000 but saw a surge in 2007, now exceeding 2,000 titles in 2014. Research focuses on stress, pain, depression and emerging interest in cancer, with systematic reviews indicating growing evidence quality [7]. The disease pattern identified in trials in our study also corroborate with this publication pattern. The research publication trained found in 2020, as reported by Gururaja *et al.* that clinical trials composes of 10-15% of the total study [13]. We found that in 2024 (cut off 10 September 2024), the percentage of trial is 14.72%. Hence, the ratio did not change to noticeable level from 2020. We found that the majority of the trials are from India and USA. This may be due to rich tradition of yoga practice in India. Mohanty *et al.* reported that female gender, Hindu religion, higher education and professional advice were linked to higher odds of yoga practice, while higher socioeconomic status was associated with lower odds. Sociodemographic factors and learning sources significantly influence continued yoga practice [14]. In USA, use of different techniques like meditation (18.3%), yoga (16.8%) and relaxation techniques (6.7%) grew significantly [15]. From physiological point of view, yoga has many benefits such as lower heart rate, blood pressure, weight loss and increased muscle strength [16]. A recent increase in interest in non-communicable disease in India also emphasize the need of more clinical trial in India [17].

## Novelty and limitation:

This study offers a comprehensive analysis of global and Indian research trends in yoga, utilizing a large dataset from the WHO ICTRP. It provides a unique overview of the distribution of yoga-related trials across different registries, highlighting India's prominent role and the increasing global interest in yoga interventions [18]. By examining the trends over two decades, the study sheds light on the growing acceptance of yoga as a therapeutic modality and identifies key areas of focus, such as chronic diseases and mental health conditions [19]. Despite its comprehensive nature, the study has some limitations. Many of the studies like observational studies are not registered, potentially skewing the results. In addition, the study does not account for variations in the quality or methodology of the trials. Furthermore, while the analysis covers a broad range of registries, it may not capture all relevant yoga-related research, particularly those from less prominent or newly established registries.

## Conclusion:

Global and Indian clinical trial research trends on yoga by examining data from the WHO ICTRP are of interest. The findings reveal a significant predominance of interventional studies, a balanced gender representation in most trials and a broad age range of participants. India stands out as a major contributor to yoga research, reflecting its cultural and historical ties to the practice. The upward trend in trial registrations over the past two decades highlights the growing global interest in exploring yoga's therapeutic potential for chronic diseases and mental health conditions. These insights offer valuable guidance for researchers and stakeholders aiming to enhance the evidence base for yoga's role in modern healthcare.

## Financial support:

This study was supported by National Institute of Naturopathy, Pune, India by paying the open access publication charges.

## Ethics:

This is an audit of data available in public domain. Hence, ethical clarence is not required.

## AI use:

The language and grammar of the manuscript was edited by ChatGPT-4o (Open AI), accessed on 15 Sept, 2024.

## Figures and Tables

**Table 1:** Number of yoga clinical trials (n = 2919) in various clinical trial registries

**Trial registry**	**Number (%)**
Department of Science and Technology	1 (0.03)
EU Clinical Trial Register	1 (0.03)
SLCTR	2 (0.07)
PACTR	5 (0.17)
CRIS	9 (0.31)
TCTR	12 (0.41)
NL-OMON	16 (0.55)
ReBec	26 (0.89)
ChiCTR	39 (1.34)
German Clinical Trials Register	39 (1.34)
ISRCTN	42 (1.44)
JPRN	71 (2.43)
ANZCTR	103 (3.53)
IRCT	146 (5)
ClinicalTrials.gov	761 (26.07)
CTRI	1646 (56.39)
SLCTR:
Sri Lanka Clinical Trials Registry, PACTR:
Pan African Clinical Trials Registry, CRIS:
Clinical Research Information Service, TCTR:
Thai Clinical Trials Registry,
NL-OMON:
Overview of Medical Research in the Netherlands, ReBec:
Brazilian Clinical Trials Registry, ChiCTR:
Chinese Clinical Trial Registry, ISRCTN:
'International Standard Randomised Controlled Trial Number, JPRN:
Japan Primary Registries Network, ANZCTR:
Australian New Zealand Clinical Trials Registry, IRCT:
Iranian Registry of Clinical Trials, CTRI:
Clinical Trials Registry - India
